# Effect of Four-in-One Optimized Emergency Nursing Procedure on Symptoms and Vital Signs of Patients with Mushroom Poisoning

**DOI:** 10.1155/2022/3387394

**Published:** 2022-03-31

**Authors:** Suling Li, Kunyu Liu, Zhenning Liu, Yu Wang

**Affiliations:** Department of Emergency Medicine, Shengjing Hospital of China Medical University, Shenyang 110004, China

## Abstract

Most members of the general public find it difficult to identify poisonous wild mushrooms, resulting in family food poisoning. Toxic mushroom poisoning can produce nausea, vomiting, abdominal pain, and other severe symptoms 30 minutes or more after ingestion that can even lead to death. Using a “four-in-one” optimized emergency nursing procedure to treat mushroom poisoning can reduce the rescue time and improve the survival rate of patients. This study aimed to analyze the influence of a “four-in-one” optimized emergency nursing procedure to treat patients with toadstool poisoning. A prospective randomized study was conducted. Sixteen cases of toadstool poisoning, corresponding to 78 patients admitted to our hospital from January 2017 to July 2020, were selected and divided into a study group and a control group of 39 cases each using a random number table. The control group was provided with routine emergency care, and the study group was given a “four-in-one” treatment that optimized the emergency care process; both groups were subjected to basic treatment + blood purification and other treatment measures, and the treatment time in the rescue room and the first blood purification time of the two groups were compared. Differences in routine blood tests, liver and kidney function indices, hospitalization time, coma time, treatment outcome, and nursing satisfaction before and after treatment were found. The treatment time and the first blood purification time of the study group were lower than those of the control group, and the difference was statistically significant (*P* < 0.05); ALT, AST, TBIL, TBA, and ALB were measured upon admission for the study and the control groups. The measured values of PT, APTT, CK, CK-MB, and BUN were compared for the two groups, but the difference in the values between the two groups was not statistically significant (*P* > 0.05); after 7 days of treatment, the ALT, TBA, and APTT indicators of the study group were lower than those of the control group, and the difference was statistically significant (*P* < 0.05); the measured values of ALT, AST, TBIL, TBA, ALB, PT, APTT, CK, CK-MB, BUN, and Scr after 7 days of treatment were significantly lower than those before treatment for both groups (*P* < 0.05). The length of stay for the study group was lower than that for the control group, and the difference was statistically significant (*P* < 0.05); the treatment efficiency was 87.18% for the study group, compared with 82.05% for the control group, but the difference was not statistically significant (*P* > 0.05). The study group rated nursing care as follows: very satisfactory, 79.49%; relatively satisfactory, 15.38%; and acceptable, 5.13%; the control group rated nursing care as follows: very satisfactory, 51.28%; relatively satisfactory, 30.77%; and acceptable, 12.82%; the results were statistically significant (*P* < 0.05). Using a “four-in-one” optimized emergency care process to treat patients with mushroom poisoning can significantly reduce the rescue room treatment time and the first blood purification time and improve nursing satisfaction, but has a limited effect on improving the treatment efficiency.

## 1. Introduction

Poisonous mushrooms frequently appear similar to edible mushrooms. Mushroom poisoning is more common for families and groups than for individuals. Once poisonous food has been ingested, the toxins contained in the food can bind to human proteins, inhibit RNA polymerase activity, and cause liver and kidney dysfunction. Multiple organ dysfunction syndrome and systemic inflammatory response syndrome occur in severe cases and eventually lead to patient death [1]. There is currently no specific antidote for mushroom poisoning, and comprehensive treatment methods, such as gastric lavage, catharsis, glucocorticoids, penicillin G, and blood purification, are mainly used [2].

Nursing is an important supplement to treatment that has a direct impact on the prognosis of patients. However, routine nursing intervention has low efficiency and is not compatible with the stress involved in emergency treatment [3]. It is necessary to optimize the management of nursing for the treatment of mushroom poisoning. A “four-in-one” optimized scheme for emergency nursing is proposed that combines four emergency nursing procedures: prehospital first aid, emergency rescue room treatment, emergency ICU treatment, and emergency ward treatment; this scheme streamlines the tasks of individual nursing units, enables treatment to proceed more smoothly, and seamlessly connects different nursing units [4]. The effect of a “four-in-one” optimized emergency nursing procedure on the treatment of patients with mushroom poisoning was explored in this study.

The rest of this paper is organized as follows: Section 2 discusses materials and methods used in this study, followed by results in Section 3.  Section 4 shows the experimental results analysis, and Section 5 concludes the paper with a summary and future research directions.

## 2. Materials and Methods

### 2.1. Information

In this study, 16 cases of mushroom poisoning, corresponding to 78 patients admitted to our hospital from August 2017 to July 2020, were selected and randomly divided into a study group and a control group with 39 cases in each group. Inclusion criteria were as follows: (1) subjects with an age range of 19–65 years; (2) epidemiological investigation showing a history of eating wild white mushrooms with similar poisoning manifestations (mainly nausea, vomiting, diarrhea, fatigue, coma, and restlessness); (3) admission to the emergency department, exhibiting clear liver and kidney dysfunction at the time of admission and having missed the optimal gastric lavage time; and (4) a research program approved by the Medical Ethics Committee. The exclusion criteria were as follows: (1) presence of malignant tumors; (2) presence of blood system diseases; (3) occurrence of multiple organ failure and disseminated intravascular coagulation (DIC); and (4) consolidation of major underlying diseases of other systems. The research tenders and related materials were issued after a decision was made by the Medical Ethics Committee, and a paper was issued (no. 201708).

### 2.2. Basic Treatment and Blood Purification

All patients were administered basic treatments, such as liver protection, stomach protection, and infection prevention, after admission, as well as blood purification treatment. Hemoperfusion, therapeutic plasma exchange, and continuous renal replacement therapy were administered according to the degree of poisoning. The replacement solution consisted of 2000 mL of fresh frozen plasma and 450–900 mL of 6.7% albumin. The replacement waste solution was discarded and replaced with an equivalent solution. Continuous renal replacement therapy (CRRT) mode, Prismaflex continuous blood purification equipment from Ludiner, Sweden, and a disposable M150 hemodialysis filter and supporting pipelines were used.

### 2.3. Nursing Methods

The control group was subjected to routine emergency nursing procedures, prehospital vomiting, gastric lavage, and catharsis in the emergency room. Venous access was established quickly, glucocorticoids, antioxidants, and other drugs were given according to the doctor's instructions, and the hemodialysis room was instructed to prepare for blood purification treatment. Blood purification treatment was performed by strictly abiding by aseptic procedures, ensuring the catheter was properly fixed, maintaining a dry catheter mouth, and using heparin sealing to ensure there was no exudation or bleeding after the end of treatment. The heparin sealing concentration was 10 ml saline plus 2 ml heparin (12500/vessel). Oral, respiratory, diet, and psychological care were provided.

The study group was subjected to the “four-in-one” optimized procedure for first-aid nursing and prehospital first aid: telephone calls were made to emergency triage nurses to guide vomiting and to inform the emergency rescue room to prepare for admission. The green channel was opened for paying fees, taking medicine, and dispensing liquid for patients with mushroom poisoning. A venous pathway was quickly established using an intravenous indwelling needle, and glucocorticoids, antioxidants, and other drugs were given according to medical orders. Venous blood was collected for laboratory testing. A telephone call was made to the emergency ICU, the patient was prepared for blood purification, and a deep venous catheter was inserted for blood purification treatment. Patients were escorted to the emergency ICU. After receiving emergency rescue room notice, items related to blood purification treatment were quickly prepared. Immediately after receiving blood purification treatment, patients were escorted back to the emergency poisoning ward. In the emergency ward, rehabilitation and oral, respiratory, diet, and psychological care were provided.

### 2.4. Evaluation Indicators and Testing Methods

The following indicators were compared for the two groups: rescue room treatment time, first blood purification time, alanine aminotransferase (ALT), aspartate aminotransferase (AST), total bilirubin (TBIL), total bile acid (TBA), albumin (ALB), prothrombin time (PT), activated partial thromboplastin time (APTT), creatine kinase (CK), creatine kinase isoenzyme (CK-MB), urea nitrogen (BUN), creatinine (Scr), hospitalization time, treatment outcome, and nursing satisfaction.

A nursing satisfaction survey of patients and their families was carried out after treatment was completed, mainly to evaluate the timeliness of reception, completion of preparation of items by emergency room nurses, cooperation among nurses, cooperation between nurses and doctors, and service attitude. Each aspect was scored out of 20 points, and the total score was 100 points. The total score was categorized into bins of ≥90 points, 80–89 points, 70–79 points, and <70 points.

A 10 ml volume of peripheral venous blood was collected before and 7 days after treatment and placed in EDTA anticoagulant tubes. The samples were centrifuged within 1 h after blood collection at 4000 r/min for 10 min. Serum ALT, AST, TBIL, TBA, ALB, CK, CK-MB, BUN, and Scr were detected by a Hitachi 7600 automatic biochemical analyzer using required reagents. A second blood sample was taken, and PT and APTT were detected by a URIIT-600 coagulation analyzer.

### 2.5. Statistical Treatment

In this study, the distribution of the measurement indices, such as ALT, AST, TBIL, TBA, ALB, PT, APTT, CK, CK-MB, BUN, and Scr, of the patients was identified as being approximately normal or normal and expressed as ($\bar{x}$ ± *s*). The *t*-test was used to compare the results of the two groups. Noncountable data were represented as percentages and compared using *χ*2 test. The Mann–Whitney *U* test was used to compare groups of graded data. Professional SPSS 21.0 software was used for data processing at the *α* = 0.05 test level.

## 3. Results

### 3.1. Comparison of General Data between the Study and Control Groups

Age, BMI, mushroom consumption, onset time, sex, and main clinical symptoms at admission were compared between the study and control groups, and the difference was not found to be statistically significant (*P* > 0.05).  Table 1 shows the comparison of general information between the study group and the control group.

### 3.2. Comparison of Rescue Room Treatment Time and First Blood Purification Time between the Study and Control Groups

The rescue room treatment time and the first blood purification time of the study group were lower than those of the control group, and the difference was statistically significant (*P* < 0.05).  Table 2 shows the comparison of the treatment time and the first blood purification time in the rescue room between the study group and the control group.

### 3.3. Comparison of Laboratory Indices between the Study and Control Groups before and after Treatment

The ALT, AST, TBIL, TBA, ALB, PT, APTT, CK, CK-MB, BUN, and Scr measured upon admission were compared between the study and control groups, and the difference was not found to be statistically significant (*P* > 0.05). After 7 days of treatment, ALT, TBA, and APTT of the study group were lower than those of the control group, and the difference was found to be statistically significant (*P* < 0.05). The levels of ALT, AST, TBIL, TBA, ALB, PT, APTT, CK, CK-MB, BUN, and Scr in the two groups after 7 days of treatment were significantly lower than those before treatment (*P* < 0.05).  Table 3 shows the comparison of laboratory indicators before and after treatment between the study and control groups.

### 3.4. Comparison of Hospitalization Time and Treatment Outcome between the Study and Control Groups

The hospitalization time of the study group was significantly lower than that of the control group, and the difference was statistically significant (*P* < 0.05). The treatment efficiency for the study group was 87.18%, compared with 82.05% for the control group, but the difference was not statistically significant (*P* > 0.05).  Table 4 shows the comparison of hospital stay and treatment outcome between the study group and the control group.

### 3.5. Comparison of Nursing Satisfaction between the Study and Control Groups

The study group rated nursing satisfaction as follows: very satisfactory, 79.49%; relatively satisfactory, 15.38%; and acceptable, 5.13%. The control group rated nursing satisfaction as follows: very satisfactory, 51.28%; relatively satisfactory, 30.77%; and acceptable, 12.82%; the results were statistically significant (*P* < 0.05).  Table 5 displays the comparison of nursing satisfaction between the study group and the control group.

## 4. The Experimental Results Analysis

Mushroom poisoning events can occur in any season. The peak incidence occurs during the rainy season in July and August, and poisoning by white umbrella, hairy umbrella, and hairy mushrooms is most common. Early mushroom poisoning can manifest as nausea, vomiting, diarrhea, and other minor symptoms, and a false healing period often causes patients to delay treatment and miss the optimal gastric lavage time [5, 6]. Many types of toxins are involved in mushroom poisoning, and the mechanism of damage to the body is complex. Damage can be categorized based on the susceptible organs as gastroenteritis, toxic hepatitis, toxic hemolysis, and neuropsychiatric damage. Early active rescue can reduce toxin absorption and block the damage of toxins to the body [7, 8]. However, it is found in practice that factors such as communication problems among various departments of medical institutions often result in prolonged treatment times, such that optimal treatment times are missed, resulting in poor prognosis of patients [9, 10]. Optimizing the emergency nursing procedure can have a positive impact on the prognosis of patients.

The four-in-one optimized emergency nursing procedure is a new emergency nursing mode. An optimized combination of four emergency nursing procedures, prehospital first aid, emergency rescue room treatment, emergency ICU treatment, and emergency ward treatment, reduces treatment time, enables treatment to be completed within the golden time, and reduces patient mortality [5, 11]. At present, the “four-in-one” optimized emergency nursing procedure has been applied to the rescue of organophosphorus pesticide poisoning, venous thromboembolism, and acute cardiovascular and cerebrovascular diseases with good results [12, 13]. Some studies have found that using the “four-in-one” emergency nursing procedure to treat poisoning patients can help improve the patient survival rate [14, 15].

In this study, the four-in-one optimized emergency nursing procedure was applied to the treatment of mushroom poisoning. It was found that the rescue room treatment time, first blood purification time, and hospitalization time were lower than those of patients who received the routine emergency nursing intervention. This result suggests that the four-in-one optimized emergency nursing procedure offers the advantages of reducing the rescue room treatment time and the first blood purification time. This result is obtained because telephone communication is employed in the “four-in-one” optimized emergency nursing procedure to strengthen cooperation and collaboration between various departments, which helps nurses in various departments in preparation and communication, while competing for valuable treatment time. The patient treatment was changed from routine blood purification to blood purification in the emergency ICU, and deep vein catheterization was performed in the emergency rescue room, which reduced the preparation time for blood purification in the emergency ICU. The green channel was opened for patients with mushroom poisoning to reduce the waiting time for payment and dispensing and receiving medication to enable patients to receive medication more quickly [16, 17]. The absolute value of the treatment efficiency of patients receiving four-in-one optimized emergency nursing intervention was found to be higher than that of patients receiving the routine emergency nursing intervention, but the difference was not significant. This result may have been obtained because the effective rate of treatment is related to other factors such as mushroom varieties and food intake. The bias introduced by an insufficient sample size may also be a factor.

## 5. Conclusion

The following indices were also measured for the two groups before and after treatment: liver function indices, ALT, AST, TBIL, TBA, and ALB; cardiac function indices, CK and CK-MB; renal function indices, BUN and Scr; and coagulation indices, PT and APTT. It was found that using the “four-in-one” optimized emergency nursing procedure to treat patients with mushroom poisoning helps reduce liver, heart, and kidney injury and protects coagulation function. This result was obtained because the proposed procedure helps reduce the rescue room treatment time and the first blood purification time and prevents toxins from damaging important organs and the coagulation system [18, 19]. The proposed procedure was also found to improve nursing satisfaction and help in building a harmonious relationship between nurses and patients.

In summary, the use of a “four-in-one” optimized emergency nursing procedure to treat patients with mushroom poisoning can significantly reduce the rescue room treatment time and the first blood purification time and improve nursing satisfaction, but has a limited effect on improving treatment efficiency.

## Figures and Tables

**Table 1:** Comparison of general information between the study group and the control group.

Normal information	Research group (*n* = 39)	Control group (*n* = 39)	*t*/*χ*^2^	*P*
Age (years)	37.5 ± 8.4	35.5 ± 7.0	1.142	0.257
BMI (kg/m^2^)	23.8 ± 2.3	23.6 ± 2.5	0.368	0.714
Toadstool consumption (g)	67.8 ± 14.3	65.1 ± 17.0	0.759	0.450
Onset time (h)	11.3 ± 4.0	10.6 ± 3.8	0.792	0.431
Gender (%)			0.867	0.352
** **Male	22(56.41)	26(66.67)		
** **Female	17(43.59)	13(33.33)		
Main clinical manifestations (%)				
** **Nausea	39(100.00)	39(100.00)	0.000	1.000
** **Vomit	36(92.31)	39(100.00)	3.120	0.077
** **Diarrhea	26(66.67)	32(82.05)	2.421	0.120
** **Fatigue	33(84.62)	30(76.92)	0.400	0.527
** **Coma	23(58.97)	18(46.15)	1.285	0.257
** **Anxious	25(64.1)	23(58.97)	0.217	0.642

**Table 2:** Comparison of the treatment time and the first blood purification time in the rescue room between the study group and the control group ($\bar{x}$ ±*s*).

Group	*n*	Emergency room treatment time (min)	First blood purification time (h)
Research group	39	17.8 ± 4.1	4.82 ± 1.10
Control group	39	22.0 ± 5.3	5.57 ± 1.42
*t*		−3.914	−2.608
*P*		0.000	0.011

**Table 3:** Comparison of laboratory indicators before and after treatment between the study and control groups (±*s*).

Index	On admission		*t*	*P*	After 7 days of treatment		*t*	*P*
	Research group (*n* = 39)	Control group (*n* = 39)			Research group (*n* = 39)	Control group (*n* = 39)		
ALT (U/L)	817.4 ± 104.3	803.5 ± 121.7	0.542	0.590	310.7 ± 66.5^*∗*^	343.7 ± 73.4^*∗*^	−2.081	0.041
AST (U/L)	711.6 ± 98.5	728.5 ± 112.8	−0.705	0.483	275.8 ± 57.1^*∗*^	283.2 ± 60.3^*∗*^	−0.556	0.580
TBIL (*μ*mol/L)	67.9 ± 12.8	70.4 ± 13.1	−0.852	0.397	29.8 ± 7.3^*∗*^	31.5 ± 6.6^*∗*^	−1.079	0.284
TBA (*μ*mol/L)	25.8 ± 6.2	27.1 ± 5.9	−0.949	0.346	16.2 ± 3.4^*∗*^	18.0 ± 4.1^*∗*^	−2.110	0.038
ALB (g/L)	39.8 ± 3.1	39.2 ± 3.6	0.789	0.433	36.1 ± 2.0^*∗*^	36.6 ± 2.4^*∗*^	−0.999	0.321
PT (s)	21.7 ± 3.2	23.0 ± 3.7	−1.660	0.101	17.4 ± 1.8^*∗*^	17.8 ± 2.2^*∗*^	−0.879	0.382
APTT (s)	35.8 ± 3.9	36.3 ± 4.2	−0.545	0.587	31.0 ± 2.4^*∗*^	32.4 ± 2.7^*∗*^	−2.420	0.018
CK (U/L)	188.5 ± 21.6	182.7 ± 23.1	1.145	0.256	165.1 ± 22.9^*∗*^	169.4 ± 24.1^*∗*^	−0.808	0.422
CK-MB (U/L)	27.5 ± 4.8	27.0 ± 4.3	0.485	0.629	22.4 ± 4.1^*∗*^	20.8 ± 4.7^*∗*^	1.602	0.113
BUN (mmol/L)	10.8 ± 2.0	11.3 ± 2.3	−1.024	0.309	8.8 ± 1.6^*∗*^	9.2 ± 2.0^*∗*^	−0.975	0.333
Scr (*μ*mol/L)	162.4 ± 18.5	166.8 ± 21.7	−0.964	0.338	122.7 ± 14.6^*∗*^	126.4 ± 16.2^*∗*^	−1.060	0.293

Note: compared with this group before treatment ^*∗*^*P* < 0.05.

**Table 4:** Comparison of hospital stay and treatment outcome between the study group and the control group.

Group	*n*	Hospital stay (d)	Treatment outcome	
			Efficient	Die
Research group	39	11.1 ± 2.0	34 (87.18)	5 (12.82)
Control group	39	12.7 ± 2.5	32 (82.05)	7 (17.95)
*t*/*χ*^2^		−3.121	0.394	
*P*		0.003	0.530	

**Table 5:** Comparison of nursing satisfaction between the study group and the control group (*n* (%)).

Group	*n*	Very satisfied	More satisfied	Generally	Dissatisfied
Research group	39	31 (79.49)	6 (15.38)	2 (5.13)	0 (0.00)
Control group	39	20 (51.28)	12 (30.77)	5 (12.82)	2 (5.13)
*Z*		−2.679			
*P*		0.007			

## Data Availability

The data used to support the findings of this study are available from the corresponding author upon request.
